# GNAQ-Regulated ZO-1 and ZO-2 Act as Tumor Suppressors by Modulating EMT Potential and Tumor-Repressive Microenvironment in Lung Cancer

**DOI:** 10.3390/ijms24108801

**Published:** 2023-05-15

**Authors:** Hyung Seok Kim, Su In Lee, Yu Rim Choi, Jiyun Kim, Jung Woo Eun, Kyoung Seob Song, Jee-Yeong Jeong

**Affiliations:** 1 Department of Biochemistry, College of Medicine, Kosin University, Seo-gu, Busan 49267, Republic of Korea; kimhs.onco@gmail.com (H.S.K.); lsi8906@gmail.com (S.I.L.); urin204@naver.com (Y.R.C.); rika524712@gmail.com (J.K.); 2 Department of Gastroenterology, School of Medicine, Ajou University, 164 World cup-ro, Yeongtong-gu, Suwon 16499, Republic of Korea; jetaimebin@gmail.com; 3 Department of Medical Science, College of Medicine, Kosin University, Seo-gu, Busan 49267, Republic of Korea; kssong@kosin.ac.kr; 4 Institute for Medical Science, College of Medicine, Kosin University, Seo-gu, Busan 49267, Republic of Korea

**Keywords:** lung cancer, tight junctions, tumor microenvironment, zonula occludens, G protein subunit alpha q

## Abstract

Epithelial-to-mesenchymal transition (EMT) plays a critical role in the development and progression of lung cancer by promoting its invasiveness and metastasis. Using integrative analyses of the public lung cancer database, we found that the expression levels of the tight junction proteins, zonula occluden (ZO)-1 and ZO-2, were lower in lung cancer tissues, including both lung adenocarcinoma and lung squamous cell carcinoma than in normal lung tissues analyzed using The Cancer Genome Atlas (TCGA). Although the ectopic expression or knockdown of ZO-1 and ZO-2 did not affect the growth of lung cancer cells, they significantly regulated cell migration and invasion. When M0 macrophages were co-cultured with ZO-1 or ZO-2 knockdown Calu-1 cells, M2-like polarization was efficiently induced. Conversely, co-culture of M0 THP-1 cells with A549 cells stably expressing ZO-1 or ZO-2 significantly reduced M2 differentiation. We also identified G protein subunit alpha q (GNAQ) as a potential ZO-1- and ZO-2-specific activator through analysis of correlated genes with the TCGA lung cancer database. Our results suggest that the GNAQ-ZO-1/2 axis may play a tumor-suppressive role in lung cancer development and progression and highlight ZO-1 and ZO-2 as key EMT- and tumor microenvironment-suppressive proteins. These findings provide new insights for the development of targeted therapies for lung cancer.

## 1. Introduction

Lung cancer is one of the deadliest diseases worldwide, and metastasis and recurrence are major factors that affect the prognosis of patients with lung cancer [1]. Despite significant advances in lung cancer treatment, the high morbidity and mortality rates remain major challenges. Therefore, an urgent need exists to identify novel therapeutic targets for lung cancer [2].

Recent studies have suggested that tight junction (TJ) proteins, such as zonula occludens (ZOs) including ZO-1, ZO-2, and ZO-3, may play important roles in cancer development and progression [3,4,5,6]. These proteins are critical components of TJs that regulate the permeability of the epithelial and endothelial cell layers [7]. During cancer development, disruption of the TJ complex can lead to changes in the cellular environment, ultimately promoting tumor invasion and metastasis [8].

To investigate the potential roles of ZO-1 and ZO-2 in lung cancer, we analyzed large cohort data from The Cancer Genome Atlas (TCGA) and Gene Expression Database for Normal and Tumor Tissues (GENT). Our analysis revealed that ZO-1 and ZO-2 were consistently downregulated in both lung adenocarcinoma (LUAD) and lung squamous cell carcinoma (LUSC) datasets. Further analysis using the Molecular Signatures Database (MsigDB) indicated that ZO-1 and ZO-2 correlated gene sets were associated with cytoskeleton organization, cell–cell junctions, polarity, and epithelial-to-mesenchymal transition (EMT).

Next, to examine the functional consequences of ZO-1 and ZO-2 in lung cancer cells, we manipulated their expression levels and found that they did not affect lung cancer cell growth but rather played a role in regulating wound healing potential, migration, and invasion. Additionally, we demonstrated that the modulation of ZO-1 and ZO-2 expression in lung cancer cells could suppress M2-like macrophage polarization, an imperative factor in the tumor microenvironment (TME) that promotes cancer progression and metastasis. This finding highlights the potential role of ZO-1 and ZO-2 as regulators of the TME.

We further explored the molecular pathways associated with ZO-1 and ZO-2 in lung cancer and identified the G protein subunit alpha q (GNAQ) as a potential interacting partner, belonging to the G protein family of signaling molecules. GNAQ is primarily involved in mediating the signaling of G protein-coupled receptors (GPCRs) that are activated by a diverse range of extracellular stimuli, such as hormones, neurotransmitters, and sensory signals [9]. Our analysis revealed a significant correlation between GNAQ expression and ZO-1 and ZO-2 downregulation in TCGA_LUAD and LUSC datasets. Further, in vitro experiments showed that GNAQ acts as an upstream regulator of ZO-1 and ZO-2 expressions.

Collectively, our findings suggest that the GNAQ-ZO-1/2 axis plays a crucial role in lung cancer progression and may provide a novel strategy for treating lung cancer and improving patient outcomes.

## 2. Results

### 2.1. ZO-1 and ZO-2 Expression Levels Are Downregulated in Lung Cancer Tissues

**Figure 1:**
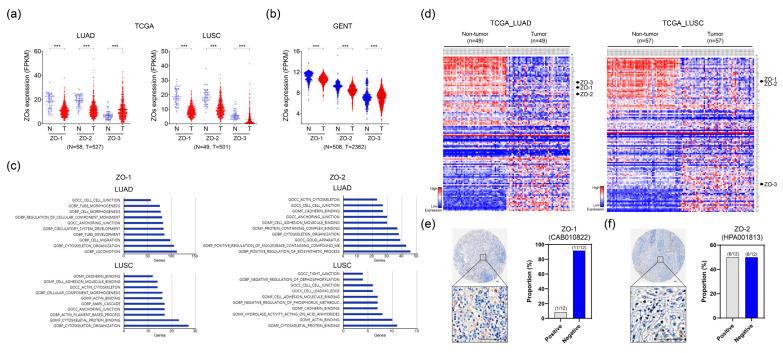
Investigation of differentially expressed ZO proteins in lung cancer. (**a**) Comparison of mRNA expression of ZOs in tumor tissues compared to non-tumor tissues in TCGA lung cancer datasets. LUAD; lung adenocarcinoma. LUSC; lung squamous cell carcinoma. (**b**) Differential gene expression of ZO mRNA in lung cancer publicly available dataset acquired by GENT2. (**c**) Gene Ontology classifications of ZO-1 (left) and ZO-2 (right) correlated genes in biological processes. (**d**) Heatmaps of differentially expressed genes associated with cell–cell adhesion or tight junctions. Left: lung adenocarcinoma (TCGA_LUAD); right: lung squamous cell carcinoma (TCGA_LUSC). (**e**,**f**) Expression of ZO-1 and ZO-2 in lung cancer tissues from Human Protein Atlas databases. Scale bars, 100 μm. *** *p* < 0.001.

ZO proteins are a family of TJ-associated proteins that regulate paracellular permeability and polarity in epithelial and endothelial cells [10]. Although the decreased expression of ZOs has been observed in various types of cancer, their role in lung cancer has not yet been fully elucidated. Here, we recapitulated the differential expression of the ZO family in TCGA_LUAD and _LUSC transcriptome data. We found that ZO-1 and ZO-2 expression levels were downregulated in patients with lung cancer from TCGA_LUAD and _LUSC (Figure 1a). To generalize this result, we analyzed the Gene Expression database of Normal and Tumor tissues (GENT2) which provides public gene expression data sets on microarrays of normal and tumor tissues [11]. In this analysis, ZO-1 and ZO-2 expression levels were also lower in lung cancer tissues than in normal tissues (Figure 1b). Although ZO-1 and ZO-2 expression levels were decreased in lung cancer tissues, a significant difference in ZO-3 expression could not be determined except in the TCGA_LUSC data set (Figure 1a,b). Next, to investigate the gene signatures enriched from known molecular databases, we acquired gene sets from MsigDB (http://software.broadinstitute.org/gsea/msgidb, accessed on 21 November 2021) at the Broad Institute Gene Set Enrichment Analysis [12,13]. There were 431 and 141 genes positively correlated with ZO-1 and ZO-2, respectively, in TCGA_LUAD, with the corresponding gene numbers of 106 and 43, respectively, in TCGA_LUSC. As expected, for the signatures of the correlated genes with ZO-1 and ZO-2 in both TCGA_LUAD and _LUSC, most gene signatures were associated with cell morphology, migration, cytoskeleton organization, and cell–cell junction identified by Gene Ontology (GO) gene set (Figure 1c). Next, we obtained a representative set of 120 genes related to TJs or cell–cell adhesion, including ZO-1, ZO-2, and ZO-3, from MsigDB. When we examined the heatmap generated from these genes, we found that the expression of cell–cell adhesion-related genes were dysregulated in lung cancer (Figure 1d). Additionally, we obtained the expression levels of ZO-1 and ZO-2 in lung cancer tissues from the Human Protein Atlas (HPA) database (https://www.proteinatlas.org/, accessed on 21 January 2022). ZO-1 and ZO-2 were mainly expressed in the membrane and showed a negative expression rate of 91.6% for ZO-1 in lung cancer tissues (Figure 1e,f). These findings indicate that the aberrantly regulated expression of TJ or cell–cell adhesion proteins has a major impact on the development and progression of lung cancer, contributing to increased cell invasiveness and metastasis.

### 2.2. ZO-1 and ZO-2 Function as Metastatic Suppressors in Lung Cancer

To elucidate the role of ZO-1 and ZO-2 in lung carcinogenesis, we performed in vitro cell growth assays. When ZO-1 or ZO-2 expression was knocked down in the lung cancer cell lines Calu-1 and NCI-H460, we observed no significant changes in cell growth and proliferation (Supplementary Figure S1a–d). Moreover, the stable overexpression of ZO-1 or ZO-2 in A549 did not affect cell growth and has similar results performed above (Supplementary Figure S1e–g).

**Figure 2:**
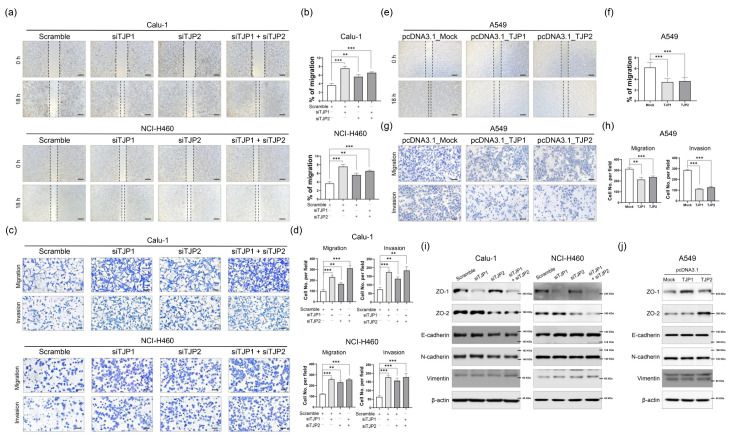
ZO-1 and ZO-2 function as metastatic suppressors in lung cancer cells. (**a**) Knockdown of ZO-1 or ZO-2 decreased cell migration analyzed with scratch wound healing assay. 100×; scale bars, 100 μm. (**b**) Scratch gap of wound healing was measured with ratio of remaining gap represented with a bar graph. (**c**) Representative images of transwell migration and invasion assays in Calu-1 and NCI-H460 200×; scale bars, 50 μm. (**d**) Bar graph represents counted number of migrated and invaded lung cancer cells. (**e**) Scratch wound healing assay was performed A549 cells stably overexpressing ZO-1 or ZO-2. 100×; scale bars, 100 μm. (**f**) Percentage of migrated cells was identified with a bar graph. (**g**) Ability of A549 cell migration and invasion was measured by modified Boyden chamber motility and transwell invasion assays. 200×; scale bars, 50 μm. (**h**) Migrated and invaded A549 cells were shown in the bar graph. (**i**,**j**) Protein expression of EMT-associated molecules was examined after transfection of siRNA targeting ZO-1 or ZO-2 in Calu-1 and NCI-H460 cells, or with A549 cells overexpressing ZO-1 or ZO-2. Scramble; Non-targeting scrambled sequence of single interference RNA (siRNA). siTJP1; TJP1 mRNA targeting siRNA. siTJP2; TJP2 mRNA targeting siRNA. pcDNA3.1_Mock; Non-expressing control vector. pcDNA3.1_TJP1; TJP1 expressing vector. pcDNA3.1_TJP2; TJP2 expressing vector. All experiments were repeated at least three times and each sample was analyzed in triplicate. ** *p* < 0.01; *** *p* < 0.001.

To clarify the roles of ZO-1 and ZO-2 in the tumor-suppressive behavior of lung cancer cells, we performed in vitro motility and invasion assays [14]. A scratch wound healing assay revealed that depletion of ZO-1 or ZO-2 expression significantly increased the wound healing efficacy in lung cancer cells (Figure 2a,b). We obtained consistent results in both the motility and invasion assays using the modified Boyden chamber assay (Figure 2c,d). Similarly, increased expression of ZO-1 or ZO-2 in A549 cells led to the suppression of metastatic potential (Figure 2e–h). To gain further insight into the regulatory effect of ZO-1 and ZO-2 on metastasis, Western blot analysis was performed to examine the EMT regulatory proteins in lung cancer cells. Not surprisingly, suppression or overexpression of ZO-1 or ZO-2 did not affect the expression of the hallmarks of EMT (Figure 2i,j). This suggests that the effects of ZO-1 and ZO-2 on migration and invasion are mediated through their roles in regulating cell–cell contact and polarity, rather than the selective regulation of EMT proteins in lung cancer.

### 2.3. ZO-1 and ZO-2 Suppresses Distinct M2 Macrophage Polarization Phenotypes

**Figure 3:**
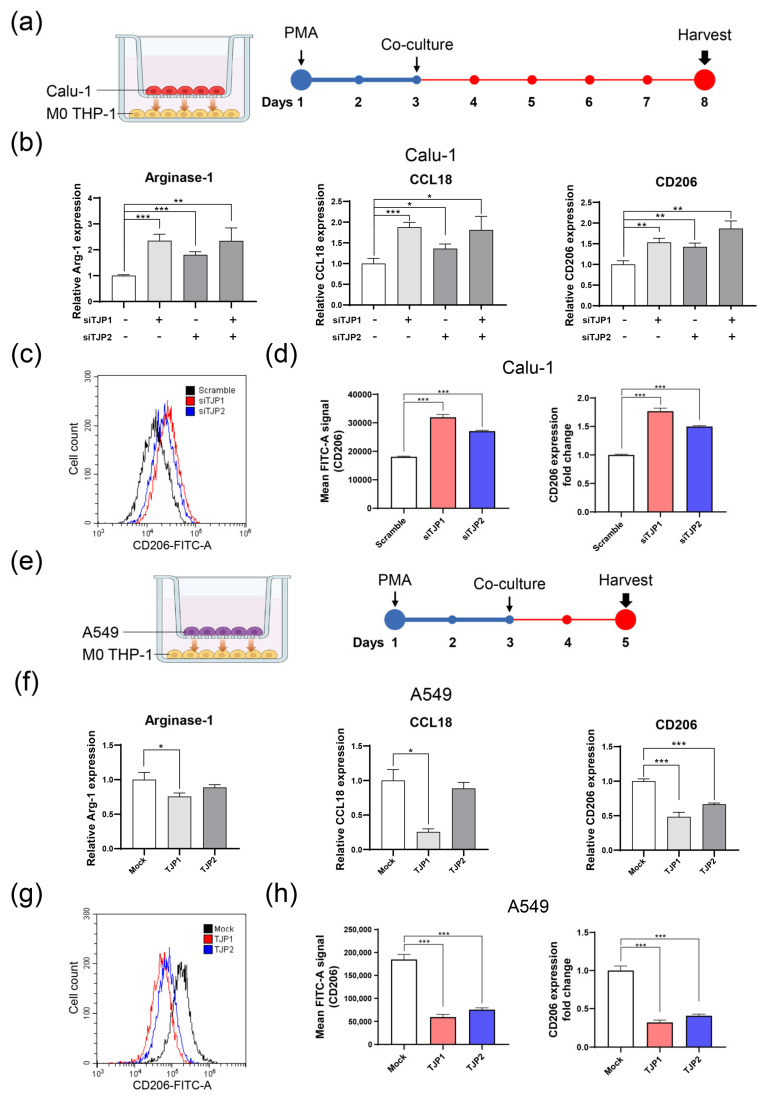
ZO-1 and ZO-2 inhibit M2-like phenotype of M0 state of macrophages. (**a**) Schematic diagram of THP-1 M2 polarization co-cultured with ZO-1 or ZO-2 knocked-down Calu-1 cells. (**b**) qRT-PCR. M2-like phenotype markers of THP-1 were examined after co-culture with ZO-1 or ZO-2 suppressed Calu-1 cells. (**c**) Histogram of CD206-positive THP-1 cells analyzed by flow cytometry analysis. (**d**) Raw peak signals of CD206-FITC (left) and their fold changes (right) are represented with bar graphs. (**e**) Schematic diagram of co-culture between A549 and M0 THP-1. (**f**) After M0 THP-1 cells were co-cultured with A549 cells stably overexpressing ZO-1 or ZO-2, M2 phenotypic markers of THP-1 were evaluated with qRT-PCR. (**g**) Flow cytometry analysis was performed to examine CD206 surface expression on THP-1 cells. (**h**) Mean FITC-A signal and expression fold change of CD206 were shown with bar graph analyzed with FACS. Scramble; Non-targeting sequence of siRNA. siTJP1; TJP1 mRNA targeting siRNA. siTJP2; TJP2 mRNA targeting siRNA. Mock; Non-expressing negative control vector (pcDNA3.1). TJP1; TJP1 expressing vector. TJP2; TJP2 expressing vector. All experiments were repeated at least three times and each sample was analyzed in triplicate. * *p* < 0.05; ** *p* < 0.01; *** *p* < 0.001.

After induction of phorbol 12-myristate 13-acetate (PMA) at a concentration of 100 ng/mL for 48 h, THP-1 cells (human monocyte cell line) became M0 macrophages. ZO-1 and ZO-2 high-expressing lung cancer cell lines, Calu-1 and NCI-H460, were co-cultured with M0 macrophages to induce sequential M2-like polarization (Figure 3a). When ZO-1 or ZO-2 knocked down Calu-1 and M0 THP-1 cells were co-cultured, the mRNA levels of M2 markers, arginase-1, CCL18, and CD206, were significantly increased (Figure 3b). To further validate this finding, we examined the surface protein expression levels of CD206 using flow cytometry (Figure 3c,d). In contrast to the results for Calu-1, THP-1 cells did not show a clear M2 polarization in response to ZO-1 or ZO-2-suppressed NCI-H460 cells (Supplementary Figure S2a–c). This is likely due to differences in the cellular context between Calu-1 and NCI-H460 cells, which could explain the different outcomes. To support the results of Calu-1, A549 cells stably overexpressing ZO-1 or ZO-2 were co-cultured with THP-1 cells in the M0 state, and changes in M2 markers were examined. Similarly, the mRNA expression levels of arginase-1, CCL18 and CD206 were significantly decreased, and CD206 protein levels were also significantly suppressed when co-cultured with A549 cells overexpressing ZO-1 or ZO-2 (Figure 3e–h). Based on these results, the aberrant changes in the expression of ZO-1 and ZO-2 during the onset and development of lung cancer suggest that they may be involved in regulating the TME.

### 2.4. GNAQ Is an Upstream Regulatory Factor of ZO-1 and ZO-2

**Figure 4:**
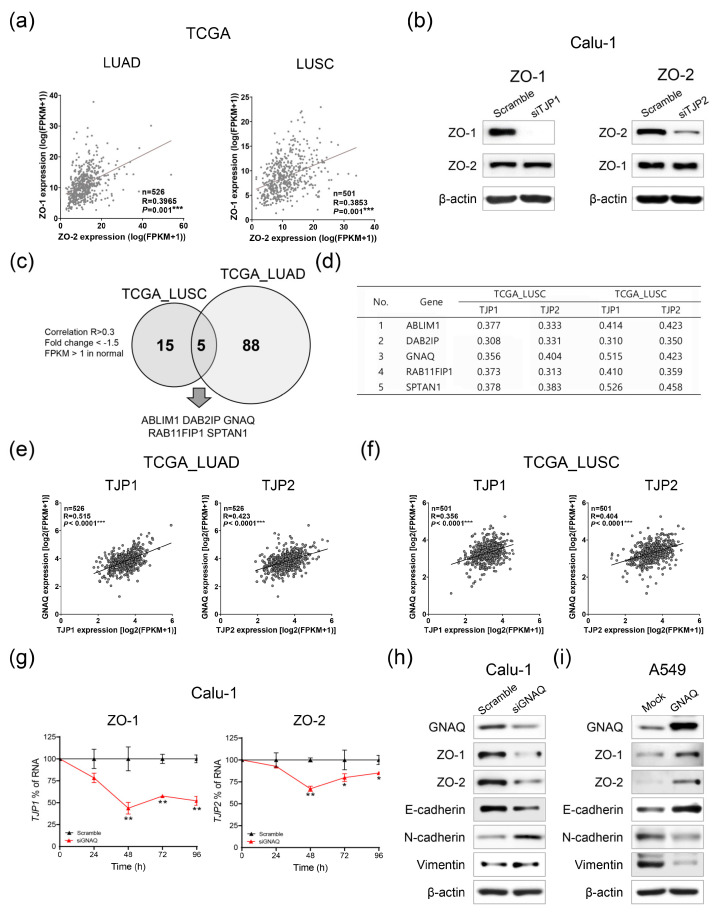
GNAQ is an upstream regulator of ZO-1 and ZO-2. (**a**) Correlation analysis of ZO-1 and ZO-2 in TCGA_LUAD and _LUSC. (**b**) Western blot analysis. Crosstalk effects between ZO-1 and ZO-2 were examined in Calu-1 cells. (**c**) Overlapping candidate genes of ZO-1 and ZO-2 regulatory targets were analyzed with TCGA_LUAD and _LUSC datasets. (**d**) Top five positively correlated genes in TCGA_LUAD and _LUSC. (**e**,**f**) Correlated scatter plots between ZO-1 or ZO-2 and GNAQ were visualized in TCGA_LUAD and _LUSC. (**g**) mRNA expression levels of ZO-1 (left) or ZO-2 (right) were examined at different time points with qRT-PCR after GNAQ knockdown in Calu-1 cells. (**h**,**i**) Protein expression of ZO-1, ZO-2 and EMT markers were investigated with Western blotting after knockdown or overexpression of GNAQ. Scramble; Non-targeting scrambled sequence of siRNA. siTJP1; TJP1 mRNA targeting siRNA. siTJP2; TJP2 mRNA targeting siRNA. siGNAQ; GNAQ mRNA targeting siRNA. All experiments were repeated at least three times and each sample was analyzed in triplicate. * *p* < 0.05; ** *p* < 0.01; *** *p* < 0.001.

To gain further insight into the inactivation mechanism of ZO-1 and ZO-2 in lung cancer, we investigated whether the expression of ZO-1 and ZO-2 is correlated with each other. Interestingly, the expression of ZO-1 and ZO-2 were significantly correlated in both TCGA_LUAD and _LUSC datasets (Figure 4a). Therefore, to investigate whether there is positive feedback crosstalk between ZO-1 and ZO-2, their expression was examined by ZO-1 or ZO-2 knockdown. However, because the depletion of ZO-1 or ZO-2 did not affect their expression levels, we assumed that they were regulated by a common upstream mechanism (Figure 4b).

To investigate the potential upstream regulators of ZO-1 and ZO-2, we analyzed the differentially expressed genes and their correlations in TCGA_LUAD and _LUSC datasets. There were 20 genes in TCGA_LUSC and 93 genes in TCGA_LUAD that were downregulated by <−1.5-fold change and commonly correlated with ZO-1 and ZO-2 (Pearson R > 0.3). Overlapping analysis between TCGA_LUAD and _LUSC showed five overlapping candidate genes that possibly regulate ZO-1 and ZO-2 as common upstream regulatory molecules (Figure 4c,d). To select specific candidate upstream molecules, we conducted a reference search and found evidence that the GNAQ gene is a tumor suppressor that inhibits EMT, especially in lung cancer [15]. We also observed a strong correlation between GNAQ and ZO-1/2 expression in TCGA_LUAD and _LUSC datasets (Figure 4d–f). To further validate this relationship, we manipulated GNAQ expression to regulate the expression of ZO-1 and ZO-2. Notably, when GNAQ was suppressed using GNAQ-targeted siRNA in Calu-1 cells, the mRNA expression levels of both ZO-1 and ZO-2 were significantly downregulated (Figure 4g). Similarly, Western blot analysis revealed a decrease in ZO-1 and ZO-2 protein levels induced by GNAQ knockdown in Calu-1 cells (Figure 4h). Conversely, overexpression of GNAQ in A549 cells resulted in increased expression of ZO-1 and ZO-2, indicating that their expression was controlled by GNAQ, an upstream regulatory molecule of ZO-1, and ZO-2 in lung cancer (Figure 4i). Note that while ZO-1 and ZO-2 efficiently suppressed the metastatic potential of lung cancer cells, there was no significant impact on the expression of EMT-associated proteins, such as E-cadherin, N-cadherin, and vimentin (Figure 2i,j). However, GNAQ sufficiently regulated these EMT hallmarks, suggesting that GNAQ not only regulates the expression of ZO-1 and ZO-2 as an upstream regulator but also acts as a suppressive regulator of EMT (Figure 4h,i). 

## 3. Discussion

ZOs are scaffolding proteins that form a framework for the assembly of multiple protein complexes on the inner side of intercellular junctions [16]. Over the past few years, there has been growing evidence indicating that ZO proteins are not only just involved in maintaining structural barriers but also play a role in signal transduction and modulation of transcriptional activity [7,16,17,18]. Considering their multiple functions, the disruption of their expression may influence the development of many diseases, such as cancer. For example, the expression of ZO-1 is significantly downregulated in liver cancer tissues compared with normal tissues, and the overexpression of ZO-1 inhibits cell migration and growth by inducing G_0_/G_1_ phase arrest [6]. ZO-2 was also previously identified as a tumor suppressor due to its structural similarities to disc-large (DLG), a known tumor suppressor protein, with 63% similarity in the amino segment containing PDZ domains, 59% similarity in the SH3 domain, and 50% similarity in the GuK domain [19]. Similarly, in gastric cancer, S100 calcium-binding protein A16 (S100A16)-mediated ZO-2 ubiquitination and degradation induce cancer progression by promoting tumor metastasis [20]. 

The present study provides compelling evidence that the TJ proteins, ZO-1 and ZO-2, play critical roles in cancer progression and may serve as potential therapeutic targets for lung cancer. The downregulation of ZO-1 and ZO-2 in both lung adenocarcinoma and squamous cell carcinoma suggests that these proteins are involved in regulating cytoskeleton organization, cell–cell junctions, polarity, and EMT. These processes are essential for maintaining the integrity of epithelial and endothelial cell layers, which are frequently disrupted in lung cancer development, leading to tumor cell invasion and metastasis [21].

The functional consequences of manipulating ZO-1 and ZO-2 expression levels in lung cancer cells were found to affect the wound healing potential, migration, and invasion, suggesting that these proteins may regulate the invasive and migratory potential of lung cancer cells. Additionally, modulation of ZO-1 and ZO-2 expression in lung cancer cells suppressed M2-like macrophage polarization, which is a critical factor in the TME that promotes cancer progression and metastasis [22].

Moreover, we identified GNAQ as a potential partner of ZO-1 and ZO-2 in lung cancer. GNAQ gene encodes a protein known as the G(q) subunit alpha, which is part of a larger complex of GTP-binding proteins that play a role in activating an enzyme called phospholipase C-beta [23]. As a result, the GNAQ protein is involved in a variety of cellular pathways, including the MAPK, PLCβ/PKC, Hippo/YAP, and PI3K/AKT/mTOR pathways [24]. However, the oncogenic or tumor-suppressive roles of GNAQ in cancer development remain controversial. For instance, the GNAQ mutation frequency is approximately 80–90% in uveal melanoma, and the p.Q209 (glutamine 209) mutation triggers a loss of GTPase activity, enabling permanent and oncogenic downstream signaling [25]. Our in silco analysis showed a significant correlation between GNAQ and ZO-1/2 expression in both lung adenocarcinoma and squamous cell carcinoma, suggesting that GNAQ is a potent upstream regulator of these two proteins. Further in vitro experimental investigation revealed that the modulated expression of GNAQ efficiently regulated ZO-1 and ZO2 expression. Moreover, the knockdown or overexpression of ZO-1 and ZO-2 did not alter levels of EMT-associated proteins, but GNAQ was found to modulate the expression of these proteins, including ZO-1 and ZO-2, suggesting that ZO-1 and ZO-2 do not exert selective regulation, but act as upstream regulators of EMT-related genes in response to GNAQ signaling. As a tumor-suppressive function, GNAQ depletion induces tumor cell growth, and metastasis from the bone to the lung promotes cancer stem cell-like properties in lung cancer cells [15]. Notably, the analysis of TCGA pan-cancer datasets revealed that GNAQ expression was downregulated in most cancer types including lung cancer.

**Figure 5:**
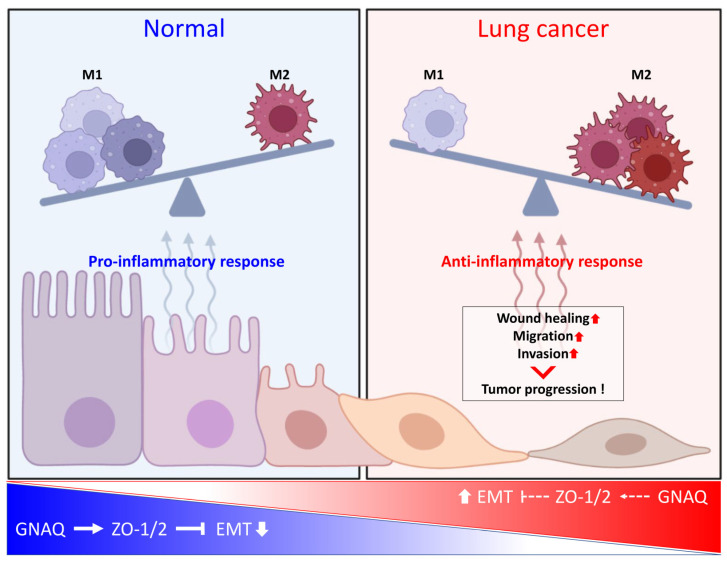
Proposed model of GNAQ-ZO-1/2 axis and its tumor suppressive function in lung carcinogenesis.

Our study provides evidence for the tumor-suppressive role of GNAQ in lung cancer and sheds light on the molecular mechanisms underlying the regulatory interactions between GNAQ and ZO-1/2. Targeting the GNAQ-ZO-1/2 axis may represent a promising therapeutic strategy for lung cancer treatment and improving patient outcomes (Figure 5). However, further research is needed to fully elucidate the molecular mechanisms underlying the interactions between these proteins, as well as to address several key questions related to the therapeutic potential of targeting this axis in lung cancer treatment. Specifically, it will be important to investigate the effect of GNAQ on macrophage polarization, the substances secreted by macrophages affected by ZO-1, ZO-2 and GNAQ-regulated lung cancer cells, and the effect of these macrophages on tumor-associated phenotypes. Most importantly, in vivo experiments using animal models will be necessary for clinical translation.

In conclusion, if these issues highlighted above are investigated and developed for clinical applications, there is a promising outlook for clinical research to utilize the findings and improve the survival and life quality of patients with lung cancer.

## 4. Materials and Methods

### 4.1. Gene Expression Profiling Using Public Databases

To assess RNA expression levels in patients with lung cancer, transcriptome sequencing data were obtained from TCGA, and GENT2 (http://gent2.appex.kr/gent2/, accessed on 4 November 2021) was used to determine gene expression patterns in lung cancer and normal tissues [11]. To identify the biological functions and molecular pathways associated with ZO-1 and ZO-2, we acquired GO gene sets from the MSigDB (https://www.gsea-msigdb.org/gsea/msigdb/, accessed on 21 November 2021) [12,13].

### 4.2. Cell Culture

Human lung cancer cell lines (Calu-1, NCI-H460 and A549) were purchased from KCLB (Korea Cell Line Bank, Seoul, Republic of Korea) and cultured in RPMI-1640 or McCoy’s 5A (Corning, New York, NY, USA) with 10% fetal bovine serum (Gibco, Billings, MT, USA), glutamine and antibiotics, and maintained in a humidified incubator with 5% CO_2_ at 37 °C.

### 4.3. Transfection and Treatment

Small interfering RNAs (siRNAs) were purchased from Bioneer (Daejeon, Republic of Korea). The sequences of siRNAs are listed in Supplementary Table S1. Transfection was performed with 100 nM of siRNA using Lipofectamine RNAiMAX (Invitrogen, Waltham, MA, USA).

Human TJP1, TJP2, and GNAQ expression plasmids subcloned into pcDNA3.1+/C-(K)-DYK plasmid were purchased from Genscript (Piscataway, NJ, USA). For expression vector transfection, 1 μg of plasmid was transfected to cells in 6-well plate with Lipofectamine 2000 reagent (Invitrogen). After 6 h of incubation, the serum free medium containing siRNA or plamid was replaced with new complete media. The transfected cells were used for further experiments at 24 or 48 h after transfection. 

### 4.4. Quantitative Real-Time PCR 

Total RNA was extracted from cells using the RNeasy Mini Kit (Qiagen, Hilden, Germany) according to the manufacturer’s protocol. cDNA was synthesized starting from 1 µg of purified total RNA using Accupower RT premix and an oligo(dT) primer (Bioneer). mRNA levels were quantified using SYBR Green (Takara, Tokyo, Japan) and an MIC qPCR Cycler system (Bio Molecular Systems, Upper Coomera, Australia). The primer sequences are listed in Supplementary Table S2.

### 4.5. Western Blot Analysis

Harvested cells were lysed in RIPA buffer (Elpis Biotech, Daejeon, Republic of Korea) supplemented with a protease inhibitor cocktail and phosphatase inhibitors (Sigma-Aldrich, St. Louis, MO, USA). A total of 10 μg of protein samples were subsequently loaded onto SDS-PAGE gels. After electrophoresis, the separated proteins were transferred onto nitrocellulose membranes (Millipore, Billerica, MA, USA). Membranes were blocked with StartingBlock (Thermo Fisher Scientific, Waltham, MA, USA) and probed with specific primary antibodies against the following: ZO-1, ZO-2, E-cadherin, N-cadherin, vimentin, and GNAQ (1:1000; all from Cell Signaling Technology, Danvers, MA, USA). A mouse primary antibody against β-actin (1:10,000; Cell Signaling Technology) was used as a loading control and to normalize the data.

The expression levels of the target proteins were determined using a chemiluminescence kit (Advansta Corp., San Jose, CA, USA) and an Amersham Imager 800 (GE Healthcare Life Sciences, Amersham, UK).

### 4.6. Proliferation Assay with Cell Counting Kit-8

Cell proliferation was measured using the Cell Counting Kit-8 (CCK-8) assay (Enzo Life Sciences, Farmingdale, NY, USA) according to the manufacturer’s protocol. After transfection of siRNAs, the cells were stained with 10 μL of CCK-8 dye in 90 μL of culture medium for 2 h at 37 °C. Absorbance was measured at 460 nm.

### 4.7. Clonogenic Assay

siRNA-transfected cells or cells stably overexpressing ZO-1 or ZO-2 were re-seeded into six-well plates and incubated for two weeks. The cells were then washed with PBS and fixed with 1% paraformaldehyde for 30 min at room temperature. Fixed cells were stained with 0.5% crystal violet for 1 h at room temperature and the stained cells were washed with distilled water. Colonies were counted using a clono-counter program [26].

### 4.8. Wound Healing Assay

The cells were trypsinized and 1 × 10^6^ cells per well were seeded in a 6-well plate. After overnight incubation, cell monolayers were scraped with a sterile 1000 μL micropipette tip. The initial gap widths at 0 h after scratching and residual gap widths at 18 h after scratching were photographed using an Eclipse Ts2 (Nikon, Tokyo, Japan).

### 4.9. Migration and Invasion Assay

For in vitro cell migration and invasion, cell motility was measured using a modified Boyden chamber assay. Matrigel (BD Biosciences, Franklin Lakes, NJ, USA) was diluted to a concentration of 0.3 mg/mL with a coating buffer for invasion assay. A total of 100 μL of Matrigel aliquots were used to coat the upper surface of the Transwell cell culture inserts (0.4 µm sized pore, Corning). After incubation for 2 h at 37 °C, the inserts were ready to be seeded with the cells. After preparation, the cells were plated on the top surfaces of the Transwell inserts, which were placed in a 24-well plate. The lower wells contained 2% FBS as a chemoattractant. The plate was incubated overnight and stained using the Diff-Quik staining kit (Sysmex, Kobe, Japan). Cell images were captured using an Eclipse Ts2 (Nikon) at 200× magnification, and the number of cells was counted in three random image fields.

### 4.10. Co-Culture Procedures for M2 Polarization

Co-culture was performed using a cell culture insert (0.4 µm sized pore, Corning). THP-1 cells (1 × 10^6^ cells/mL) were seeded in a 24-well plate and treated with 100 ng/mL PMA (Sigma-Aldrich, St. Louis, MO, USA) for 48 h to stimulate M0 differentiation. Differentiated cells were rinsed with PBS and Calu-1, NCI-H460 and A549 were seeded in the upper chamber of the insert. Calu-1 and NCI-H460 cells were incubated for 5 days and A549 cells were incubated for 2 days with M0 THP-1 cells. To confirm the differentiation of M2-like THP-1 cells, qRT-PCR and flow cytometry were performed to examine changes in the expression of differentiation markers.

### 4.11. Macrophage Phenotypic Analysis by Flow Cytometry

THP-1 cells were tested for cell surface antigen expression using flow cytometry. The cells were harvested and rinsed with PBS, after which CD206-FITC (Miltenyi Biotec, Bergisch Gladbach, Germany) and 7-AAD (eBioscience, Inc., San Diego, CA, USA) were added. Thereafter, the cells were incubated at 4 °C for 30 min in the dark. The stained cells were analyzed using CytoFLEX (Beckman Coulter Inc., Brea, CA, USA).

### 4.12. Statistical Analysis

All experiments were performed at least three times and all samples were analyzed in triplicate. Differences between the groups were analyzed using a paired *t*-test for multiple comparisons using GraphPad Prism software (version 8.0; GraphPad Inc., San Diego, CA, USA), and the quantitative assays were presented as means ± standard deviation. *p* < 0.05 was statistically significant and asterisks were used to indicate different levels of significance. 

## 5. Conclusions

The present study highlights the critical roles of ZO-1 and ZO-2 in lung cancer progression. These findings provide a valuable starting point for the development of new therapeutic strategies against lung cancer. Although this study revealed promising results, further research is required to fully comprehend the mechanism underlying the GNAQ-ZO-1/2 axis and explore its potential as a therapeutic target for lung cancer treatment. Such investigations can have significant implications for improving the prognosis of patients with lung cancer and may ultimately lead to the development of more effective treatment options for lung cancer.

## Data Availability

The data for this study are available from the corresponding author upon reasonable request.

## Supplementary Materials

The following supporting information can be downloaded at: https://www.mdpi.com/article/10.3390/ijms24108801/s1.
