# Supplementary material for: GNAQ-Regulated ZO-1 and ZO-2 Act as Tumor Suppressors by Modulating EMT Potential and Tumor-Repressive Microenvironment in Lung Cancer
Source: Int J Mol Sci. 2023 May 15;24(10):8801. doi: 10.3390/ijms24108801 (PMC10218451; doi:10.3390/ijms24108801)
Supplement: Supplementary file 1 [file ijms-24-08801-s001.zip › ijms-2385858-supplementary.pdf]

# GNAQ-regulated ZO-1 and ZO-2 Act as Tumor Suppressors by Inhibiting EMT Potential and Tumor-repressive Microenvironment in Lung Cancer

**Supplementary Materials**

**Supplementary Table S1.** List of siRNAs used in transfection.

| siRNA/miRNA | Strand | Nucleotide sequence |
| --- | --- | --- |
| Control siRNA | Sense | 5'-CCUACGCCACCAAUUUCGU-3' |
| | Antisense | 5'-ACGAAAUUGGUGGCGUAGG-3' |
| TJP1 siRNA | Sense | 5'-GGAUAGAAGUGCAAGUAGA-3' |
| | Antisense | 5'-UCUACUUGCACUUCUAUCC-3' |
| TJP2 siRNA | Sense | 5'-GACAAGGUGUCAAAACCAU-3' |
| | Antisense | 5'-AUGGUUUUGACACCUUGUC-3' |
| GNAQ siRNA | Sense | 5'-AGUGUACCAGUUUACAGAU-3' |
| | Antisense | 5'-AUCUGUAAACUGGUACACU-3' |

**Supplementary Table S2.** List of primer sequences used in qRT-PCR experiments.

| Gene | Primer | Nucleotide sequence |
|---|---|---|
| TJP1 | Forward | 5'-CCAGAAATACCTGACGGTGC-3' |
| | Reverse | 5'-TGTCTGTGCTCATAGCCTTG-3' |
| TJP2 | Forward | 5'-CAAAACCATTCTCACAGCCCC-3' |
| | Reverse | 5'-CCGGACACTGCAATTCCAAA-3' |
| Arg-1 | Forward | 5'-CTTGGCAAAAGACTTATCCTTAG-3' |
| | Reverse | 5'-ATGACATGGACACATAGTACCTTTC-3' |
| CCL18 | Forward | 5'-TGGCAGATTCCACAAAAGTTCA-3' |
| | Reverse | 5'-GGATGACACCTGGCTTGGG-3' |
| CD206 | Forward | 5'-TGAATTGTACTGGTCTGTCCT-3' |
| | Reverse | 5'-CTGTGGTGCTGTGCATTTATCT-3' |
| GNAQ | Forward | 5'-TGGAGTCCATCATGGCGTG-3' |
| | Reverse | 5'-CACTCTCTCCTGTCCCGAGC-3' |
| β-actin | Forward | 5'-GCCAACAGAGAGAAGATGACAC-3' |
| | Reverse | 5'-GTAACACCATCACCAGAGTCCA-3' |

**Supplementary Figures**

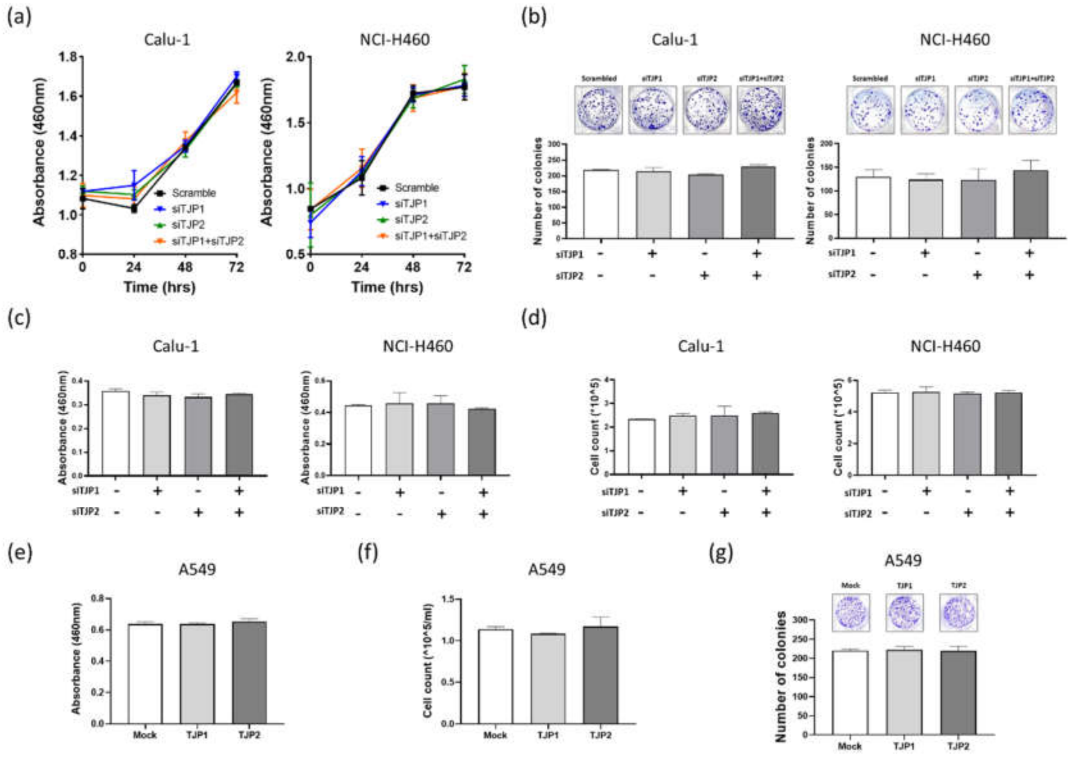


**Supplementary Figure S1.** ZO-1 and ZO-2 do not affect the cell proliferation of lung cancer cells (a) The cell growth was examined at different time points measured by Cell Counting Kit-8 assay at the absorbance of 450 nm after knockdown of ZO-1 or ZO-2. (b) The growth of lung cancer cells was determined by clonogenic assay at 72 h. (c) The lung cancer cells' viability was examined using Cell Counting Kit-8 at 72 h. The absorbance at 450 nm is presented. (d) Cell viability was assessed by counting the viable cells at 72 h. (e,f) The cell proliferation or viability of ZO-1 or ZO-2 overexpressing A549 cells was determined by Cell Counting Kit-8 or a cell counter at 72 h. (g) Clonogenic assay and quantification of ZO-1 or ZO-2 overexpressing A549 cells, cultured over 2 weeks, followed by crystal violet staining.

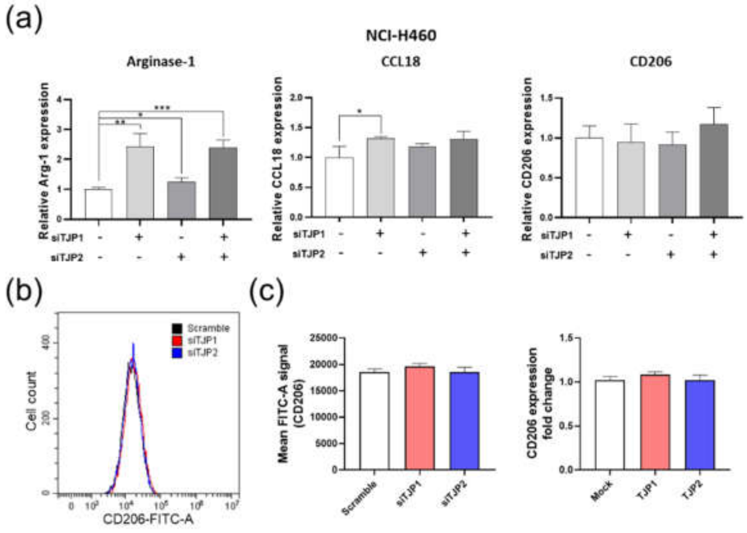


**Supplementary Figure S2.** M2-like phenotypic polarization of THP-1 was not affected by NCI-H460 cells (a) M2 phenotype markers were assessed by qRT-PCR after co-culture with ZO-1 or ZO-2 repressed NCI-H460. (b) Histogram of CD206 expression analyzed by flow cytometry. (c) Raw peak signals of CD206-FITC (left) and their fold changes (right) are shown in the bar graphs.
